# *Ctenolepisma longicaudatum* Escherich (1905) Became a Common Pest in Europe: Case Studies from Czechia and the United Kingdom

**DOI:** 10.3390/insects12090810

**Published:** 2021-09-10

**Authors:** Martin Kulma, Terezie Bubová, Matthew Paul Davies, Federica Boiocchi, Jiří Patoka

**Affiliations:** 1 National Reference Laboratory of Vector Control, National Institute of Public Health, Šrobárova 49/48, 100 00 Prague, Czech Republic; terezie.bubova@szu.cz; 2 Department of Zoology and Fisheries, Czech University of Life Sciences Prague, Kamýcká 129, 165 00 Prague, Czech Republic; patoka@af.czu.cz; 3 Killgerm Chemicals Ltd., Wakefield Road, Ossett WF5 9AJ, UK; matthew.davies@killgerm.com; 4 Department of Food, Environmental and Nutritional Sciences (DeFENS), University of Milan, Via Celoria, 2, 20133 Milan, Italy; federica.boiocchi@unimi.it

**Keywords:** silverfish, spread, invasive species, synanthropic species, pest, Lepismatidae, Europe

## Abstract

**Simple Summary:**

*Ctenolepisma longicaudatum*, an invasive silverfish species, has high tolerance to low humidity and temperatures and has been passively introduced to the majority of European territory. Its presence may cause stress and discomfort to people inhabiting or working the infested areas. In addition, it may cause damage to organic materials, contaminate food and carry microbes. This paper summarizes the available data on its spreading and current distribution of species in Europe and displayed the species is present throughout the continent including Scandinavia. This northward shift of *C. longicaudatum* might be explained by use of the insulation and central heating to provide optimal climate for the species to establish. The paper also contains updates on the current status of *C. longicaudatum* in Czechia, United Kingdom, and Ireland, where its first populations were recently detected. Based on the collected data, the spread in the monitored countries continues rapidly, when the domestic settings were the main habitat. Furthermore, the species is often present in accommodation facilities, warehouses, factories, public institutions, shopping malls, archives, museums, and art galleries. Therefore, the study indicates the species may occur everywhere indoors. The paper also highlights an urgent need for establishment of effective pest management strategy and preventive measures.

**Abstract:**

Synanthropic invasive silverfish, *Ctenolepisma longicaudatum*, has been recently reported to cause nuisance in the indoor environment in many European countries. To get more details on the species distribution, the species occurrence was monitored by the authors in the countries where establishment of *C. longicaudatum* has been revealed in the last years. In Czechia, 20 findings from 14 municipalities in eight regions were recorded within the last three years. In the United Kingdom, 49 cases, including the first occurrence in Scotland, were recorded. Five cases were recorded for the Republic of Ireland. Domestic settings were the main habitat in the study countries (50.0% for the Czechia and Ireland and 36.8% for the United Kingdom). Regarding *C. longicaudatum* control, the standard silverfish strategy fails, and the use of insecticidal baits complemented by dust insecticides was suggested as the most promising approach. To reveal presence of *C. longicaudatum* in Europe, the search of literature, social platforms and databases on invasive species was conducted. According to these sources, the species is known from majority of European countries, when the high increase of records in recent decade was detected.

## 1. Introduction

Biological invasions, in general, cause huge global environmental and socio-economic losses [1], when intercontinental traffic is known to be one of the main sources of non-native organisms [2] and anthropogenic activities are the main driver of non-native species introductions. Therefore, the synanthropic species can become invaders in human infrastructure and urban environments, even if their survival in wildlands may be limited by local climatic conditions [3].

*Ctenolepisma longicaudatum* Escherich (1905) (Zygentoma; Lepismatidae), a wingless primitive insect, whose adults can live for a long time and continue to molt. The species also known under the common names of giant silverfish, gray silverfish, long-tailed silverfish, or paperfish [4,5] is a synanthropic invasive pest from the family Lepismatidae, with uncertain native range [6,7], even though some authors speculate that it may originate from the central parts of America [7]. The species was described by the German entomologist and professor of zoology Karl Leopold Escherich in 1905 based on type material collected in the Orange Free State, Republic of South Africa [8]. Recently, the gender of the genus was shifted from feminine to neuter, and so, the species name should be changed from *longicaudata* to *longicaudatum* [9]. The species was synonymized with *Ctenolepisma coreana* Uchida (1943), *Ctenolepisma dives* Silvestri (1908), *Ctenolepisma pinicola* Uchida (1964), *Ctenolepisma urbana* Slabaugh (1940), and *Lepisma corticola* Ridley (1890).

Apart from other European synanthropic silverfish species, *C. longicaudatum* can survive long periods of starvation [4] and tolerate low indoor humidity and temperature levels [10]. The development time in optimal conditions is one and a half years, while it could also reach for up to three years [11]. Then, the lifespan of this silverfish has been reported to be up to seven years [12]. Therefore, it has the potential to inconspicuously become a dominant pest in the indoor environment in future years.

Even though *C. longicaudatum* is mainly nuisance in homes and pest in museums and archives, some attention from public health point of view has been also paid to silverfish activity. Generally, the crawling insects, including silverfish, are known to be sources of inhalant allergens [13]. From this point of view, tropomyosin was identified as the major allergen of *Lepisma saccharinum* by Barletta et al. [14], and its presence in other silverfish species is thus probable. Furthermore, several gregarines were found in the intestines of various silverfish species, with *Gregarina ctenolepismae* and *Lepismatophila ctenolepismae* associated with *C. longicaudatum* [15]. Recently, an association of opportunistic bacteria with *C. longicaudatum* has been reported in unpublished data of F. Boiocchi. Indeed, bacteria of the genus *Staphylococcus*, *Acinetobacter*, *Bacillus* and *Kocuria* were isolated from both the exoskeleton and internal structures of specimens of *C. longicaudatum*. Being this insect species commonly found within human dwellings, the carriage of opportunistic pathogens could represent a risk for human health. Despite a direct transmission from the insect to a human host has never been recorded, it is plausible to assume that these insects could mechanically disperse across the household bacterial cells able to cause infections in humans. This risk would be higher for immunocompromised people. Furthermore, pores and mycelial fragments of the fungi were proved to be transmitted by *C. longicaudatum* [16]. As aggregations of *C. longicaudatum* usually appear close to their sources [17], food contamination may potentially be another risk factor for humans. Nevertheless, no evidence supporting this hypothesis is currently available. The activity of such pests may generate stress and discomfort to people inhabiting or working in infested buildings [18]. Moreover, the species is considered to be an economic pest [19] due to its feeding on paper, book bindings, books, wallpaper, papier-mâché, photos and plant-based materials such as cellulose and starch [20,21]. In addition, the species can damage fabrics, especially cotton [22,23]. For these reasons, it is listed as a pest among museums and gallery with a possible devastating impact on art paintings, books, and documents [24] and where the damage costs might be incalculable. The costs of damage could be especially high when occurring in heritage sites. For instance, *C. longicaudatum* was also found on board historic ships in the United Kingdom [25].

Although the presence of *C. longicaudatum* has already been reported throughout Europe [11,26,27,28], the current range and distribution of this species is considered to be underestimated due to its cryptic behavior, likely misidentification when found and low awareness of the importance of reporting the species occurrence. Therefore, we aimed to present a review of the current status of *C. longicaudatum* across Europe and to gather information about its occurrence in the Czechia and the United Kingdom three and six years after the first records there of a case of spread of this synanthropic species using citizen science and social media.

## 2. Materials and Methods

### 2.1. Data Collection

The records of *C. longicaudatum* in Europe were collected from the publicly available on-line databases and social platforms (see Table 1). All data from databases were filtered using the categories “species” and “country”.

In Czechia, citizen and pest control service reports of the species were collected and documented by entomologists of the National Institute of Public Health in Prague (NIPH). Moreover, the group objected to the determination of invertebrates on Facebook (https://www.facebook.com/groups/bezobratli, accessed on 29 March 2021) was regularly checked for the presence of suspicious pictures.

In case of the United Kingdom, the records from internal database of Killgerm Chemicals Ltd. insect identification service (https://www.killgerm.com/technical/insect-identification/, accessed on 22 March 2021) and from a 12-month citizen science project carried out in Birmingham (UK), which surveyed the complete household arthropod community, were included. Furthermore, the literature research was performed using online databases, Web of Science, Scopus, and Google Scholar.

### 2.2. Case Regions

#### 2.2.1. Czechia

**Figure 1:**
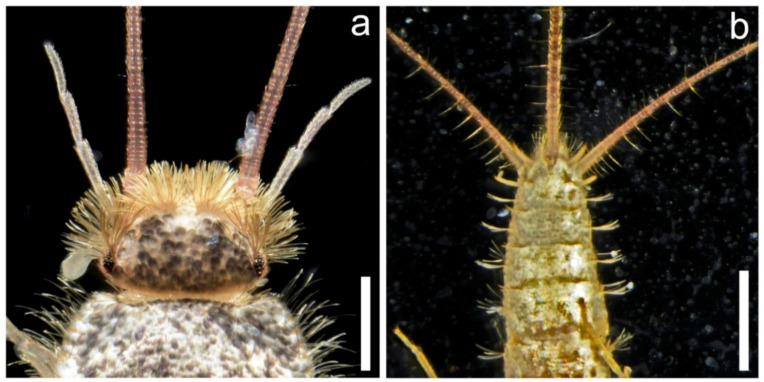
(**a**) Head of the captured male of *Ctenolepisma longicaudatum*, dorsal side; (**b**) abdomen of the captured *C.* *longicaudatum*, ventral side, (scales: (**a**) 0.1 mm; (**b**) 2 mm).

In this study, we aimed to gather information on the occurrence of *C. longicaudatum* throughout Czechia, three years after its first record [26]. The issues of this species invasion and the potential consequences were presented to both academics and professionals using various conferences and local journals, in order to increase awareness and prevent further spread. For the purpose of this study, only photos with clear morphological patterns were used. Selected localities were also inspected personally by employees of the NIPH. The captured specimens (see Figure 1) were determined using entomological keys [6,29], euthanized, and deposited at the National Reference Laboratory of Vector Control at NIPH.

#### 2.2.2. United Kingdom and the Republic of Ireland

In the United Kingdom and the Republic of Ireland, the first record of *C. longicaudatum*, in a domestic premise, was from 2014 in Reading, England [30]. Killgerm records of *C. longicaudatum* are provided from, in most cases, insect identification of physical specimens. The remainder was obtained from photographs with clear morphological patterns. The identifications were undertaken by entomologists from the Technical Department of Killgerm Chemicals Ltd. Regarding the citizen science project, volunteers in the Birmingham area were recruited and trained to carry out arthropod collection in their homes using sticky traps for crawling insects and a pooter and spider catcher for active capture. The volunteers collected household arthropods across 12 months, from November 2018 to October 2019, providing a record of the arthropods present within the households. The captures were stored in a domestic freezer (active collection) or in a sticky trap until monthly collection, and the species were then identified by an entomologist. The aim of the citizen science project was to investigate the household arthropods community in the Birmingham area, but also to explore the possible microbiological risks associated with them. Therefore, after the identification, the insects were analysed with microbiological techniques in order to isolate bacterial strains and assess the potential health hazard represented by these arthropods.

## 3. Results

**Figure 2:**
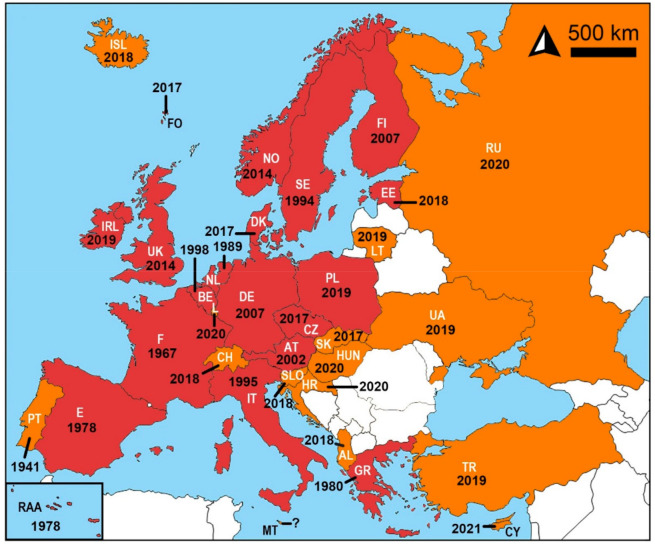
The distribution of *Ctenolepisma longicaudatum* in Europe; red: confirmed records; and orange: unverified records. The distribution data from white countries are not available/were not found among the sources.

Based on searches of selected databases and in addition to previously published and confirmed occurrences, the vast majority of the European territory was considered to have been invaded by *C. longicaudatum* (Figure 2). The oldest verified record is from 1978 in Spain, and the oldest unpublished record is from 1941 in Portugal. Various European countries and regions, including Czechia and the United Kingdom, have been invaded in recent years. By 1999, the species officially occurred in seven countries, mainly from western and southern Europe. In 2000–2021, evidence of *C. longicaudatum* is available throughout Europe, except in a few eastern European countries (Belarus, Bosnia and Herzegovina, Bulgaria, Kosovo, Latvia, Moldova, Montenegro, North Macedonia, Romania, and Serbia) and mini-states such as Andorra, Liechtenstein, Monaco, San Marino, and the Vatican.

**Figure 3:**
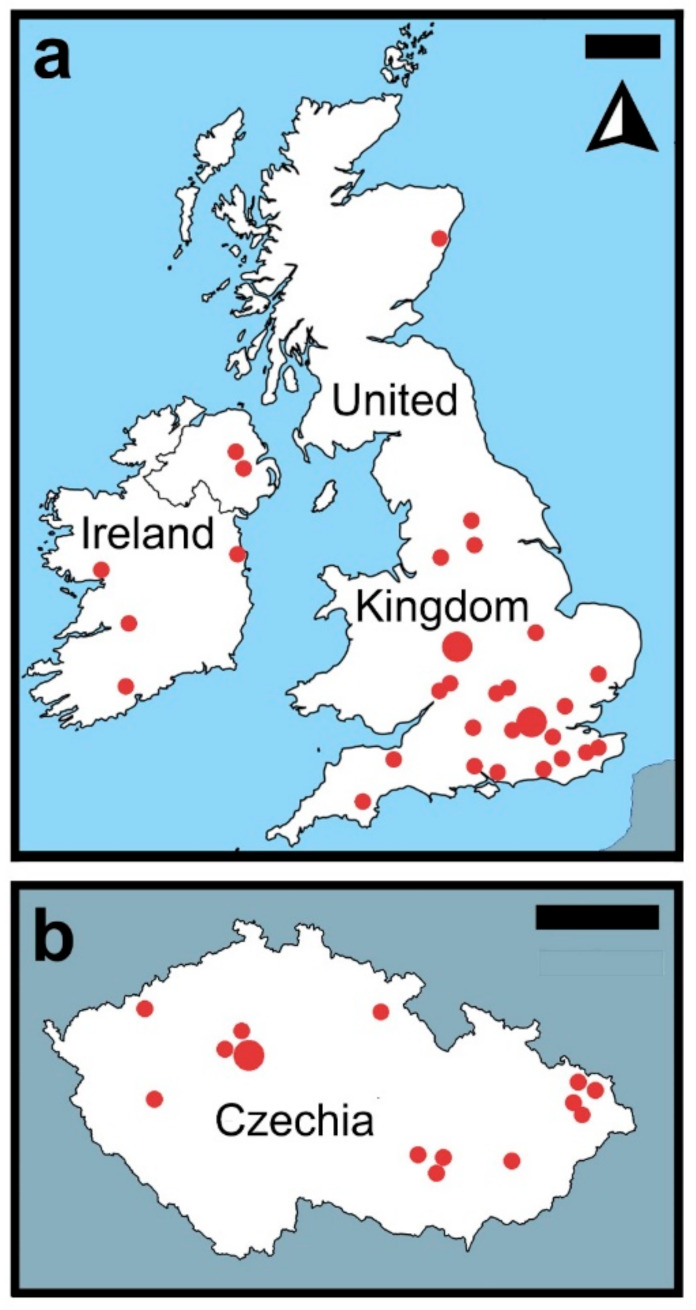
The distribution of *Ctenolepisma longicaudatum* in the United Kingdom, and Ireland (**a**) and in Czechia (**b**). Sources: iNaturalist, Observation.org, What’s Eating Your Collection, the Killgerm database and collected citizen reports.

Regarding *C. longicaudatum* in Czechia, 20 confirmed reports of the species from 14 municipalities in eight regions (Table 2 and Figure 3a) were collected by March 2021. Prague (7) had the highest number of invasions, followed by Moravian-Silesian (4), South Moravian (2), and Central Bohemian (2) regions.

From October 2017 to January 2021, Killgerm reported records from England (14), Scotland (1), and the Republic of Ireland (2). Furthermore, the citizen science research showed the species is present in households in Birmingham (England) from October 2018 to November 2019. A total of 96 individuals of *C. longicaudatum* were recovered from six out of 20 households surveyed during the year of collection. The National Biodiversity Network records show cases in Northern Ireland (3) from 2016 to 2018. Further records were added from the available social platforms and databases (Figure 3b and Table 3).

**Figure 4:**
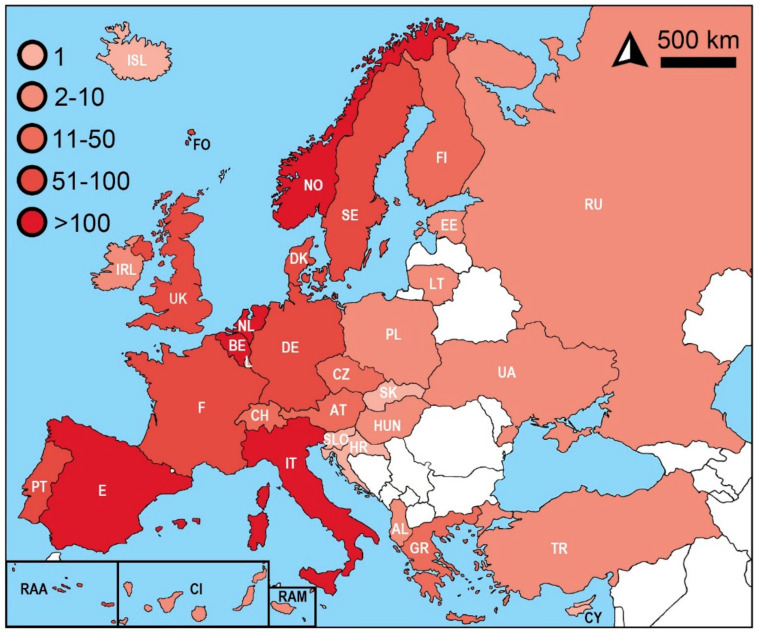
The abundance of *Ctenolepisma longicaudatum* in Europe based on citizen reports. Sources: Fauna Europea, Global Biodiversity Information Facility (GBIF), iNaturalist, Killgerm, European Network on Invasive Alien Species (NOBANIS), Observation.org, and Pan-European Species Directories Infrastructure (PESI); “What’s Eating Your Collection”, and available literature sources (see Table 4).

As shown in Figure 4 and Table 4, the highest abundance of records was found in Norway, where the pest control services reported more than 5000 interventions against *C.* *longicaudatum*. More than 100 cases of gray silverfish have also been documented in Belgium, Netherlands, Spain, and Italy. More than 50 observations were reported from Azores, Denmark, France, Germany, Portugal mainland, Spain mainland, and Sweden. On the other hand, only a single occurrence of the species is known from Iceland, Croatia (Brać Island), Slovakia, and Slovenia. The single record of the species is known from Cyprus; however, the two other surveyed databases reported the presence of *C. longicaudatum*.

## 4. Discussion

The assumption that the current distribution of *C. longicaudatum* in Europe is underestimated was confirmed, when the study revealed growing number of reports throughout the continent from scientific literature, databases of invasive species and social platforms. Although the identification of silverfish from photographs might be sometimes confusing, *C. longicaudatum* is as different from another European synanthropic species as such identification is possible [27] and the majority of the reports uploaded on the investigated social sites seem to be identified correctly. Anyway, the further collaboration among local entomologists, citizens and pest control services is essential to confirm the distribution area of the species. Additionally, the data collected on social networks indicated that *C. longicaudatum* is present (and sometimes even abundant) in countries without published reports of *C. longicaudatum.* The widespread presence of this silverfish was also supported by reports of new localities in countries with known occurrence of the species. Apparently, the frequency of new introductions has been rising in recent years, and further spreading is thus expected. The species is strictly synanthropic and does not occur outdoors; therefore, its spread is not limited by climate. Thus, it has recently become well established, even in northern Europe. For instance, in Norway, the species was officially registered in 2014, and the number of reported infestations escalated from nine to 3433 cases in 2018 [18]. From this point of view, the confirmation of gray silverfish establishments in the up-to-date non-colonized countries are expected very soon. In particular, considering the fact that some of those states are surrounded by areas with common *C.* *longicaudatum* nuisance, confirmed cases are rather the tip of the iceberg. It is also possible that the species is present but unrevealed in these areas. In the Faroe Islands, the occurrence of this silverfish pest was not known in 2019, when it was revealed in the Facebook survey conducted by Thomsen et al. [27] that *C. longicaudatum* is widespread in private houses as well as in public institutions. This is in agreement with the citizen science study from Birmingham, where almost one-third of the monitored sites were proven to be infested by *C. longicaudatum*. From this point of view, social platforms and citizen science appear to be useful tools to monitor species distribution. On the other hand, the sampling programs should be conducted to reveal the real status of the species in the countries with no confirmed presence of *C. longicaudatum*. For instance, some of them such as Andorra, Liechtenstein, Monaco, San Marino, and Vatican are surrounded by the countries where the species has been reported abundant for years, and so, their unregistered occurrence there is therefore highly probable.

*Ctenolepisma longicaudatum* is not exploited by humans and is transported unintentionally as an unseen invader; thus, even if reasonable, it is not possible to simply ban the import of the species. Therefore, its inclusion in the updated list of invasive alien species of European Union concern (Regulation 1143/2014; the so-called Union list) is likely to be totally ineffective. The higher effectiveness of any regulation is not related to increasing knowledge of the general public, as in the case of ornamentals and pets [44]. The focus should be on traders and conveyers who would be trained in mandatory preventive timely treatment of cardboard boxes and similar materials before transport.

In Czechia, the first establishment of the *C. longicaudatum* population was reported in warehouses and surrounding office buildings in 2017 [26]. Subsequently, within the next three years, the presence of *C. longicaudatum* was recorded throughout the country. As expected, the flats (nine records) were found to be the main habitats of the species. In contrast, five records of *C. longicaudatum* were obtained from warehouses. Interestingly, repeated introductions have been reported in the warehouse of a pharmaceutical company in Prague, the capital of Czechia. In 2019, one individual and several exuviae (n < 10) were found in barrels imported from Japan (Sejkora R., pers. comm. 2019), where *C. longicaudatum* was established in the 1990s [45]. Although museums, libraries, and archives were mentioned as the main habitat of *C. longicaudatum* in the past [33,46], only one such case was reported in Czechia. In 2018, the presence of *C. longicaudatum* was revealed by the National Gallery Prague. Fortunately, the staff detected the pest activity relatively soon, when the damage was found on the reverse side of the exhibited paintings where the glue was applied. No serious damage to the paintings was observed. Both room and artworks were then treated with biocides. Despite the early successful eradication, this finding is alarming and underlines previous notes on art collections caused by *C. longicaudatum*. Moreover, the lack of maintenance during shutdowns of connected to COVID-19 pandemic may result in exponential development of the silverfish populations in such institutions [47].

In the United Kingdom, *C. longicaudatum* has especially been detected in the domestic environment, but findings of the “What’s Eating your Collection” and “iNaturalist” databases revealed that the species is often present in accommodation facilities, archives, museums, and warehouses. The first evidence of *C. longicaudatum* in Scotland came from Aberdeen (Table 4), and it was suspected to have been introduced from Norway, via international travel by the resident (G. MacKay., pers. comm. 2021), according to discussions with the pest control operator. Regarding the report from a museum, the background of only a single event from southeast London (Table 4) is known, and the pest control operator reported *C. longicaudatum* among historical papers/books, paper goods, and clothing, but no damage was observed. In Ireland, two findings of the species in a domestic setting were complemented by one silverfish report from the medical center and two unknown localities (Table 4).

The novel data from the studied countries confirmed households as a major habitat of *C. longicaudatum*. This phenomenon is known, for instance, in the Faroe Islands, the Netherlands, and Norway, where the species has become a major household species [17,23,27,48]. Additionally, the inhabitants of houses are often unaware of their occurrence due to the hidden activity of silverfish pests. For instance, Witteman et al. [49] reported many homes in which the inhabitants were unaware of the presence of silverfish to contain silverfish antigen concentrations indicative of silverfish exposure. Therefore, the real levels of activity might be even higher than expected. The hypothesis of spreading using cardboard boxes and industrial, manufacturing, and warehouse facilities as one of the primary habitats suggested by Kulma et al. [26] was also supported by records of such species in these facilities in several regions throughout both countries.

The increasing intensity of records throughout the case regions highlighted the urgent need for reliable pest management. The authors recommended eradication at all the investigated localities. Based on the feedback provided by the companies in Czechia, the pyrethroids applied by spraying or smoke generator by the professional control services reduced silverfish abundance for a few months. However, the re-emergence of this species was reported by the employees of infested institutions. The species is considered to be sensitive to conventional pesticides [18,50], but the regular application of biocides containing similar active substances is not recommended because of the risk of resistance development [51]. Moreover, the suitability of commonly used insect control programmes of Integrated Pest Management (IPM) such as nitrogen or anoxia fumigation against giant silverfish due to hidden life is questionable. In view of this, novel approaches to control *C. longicaudatum* have recently received some attention. For instance, Wilke et al. [24] recommended a warm air eradication technology. The local use of heat treatment in cavities might also be beneficial according to Aak et al. [18], who also pointed out that *C. longicaudatum* inhabits spots inside walls, where the heat hardly penetrates. In light of this, the use of poisoned baits seems to be the most promising strategy. The laboratory bioassay conducted by Aak et al. [17] showed that baits with indoxacarb, clothianidin, and fipronil killed more than 90% of the insects; however, high secondary toxicity was observed in indoxacarb bait only (>75%). Therefore, same authors [52] also verified the effect of such a substance in the field and reached >90% population decline with minimal bait consumption. Therefore, using baits containing indoxacarb might be an economic and efficient strategy against this target species. Similarly, a field test in the Netherlands revealed that baits containing clothianidin caused a 93% population reduction [53]. Aak et al. [51] also highlighted that proper training in bait placement and improved knowledge of *C.* *longicaudatum* bionomy is crucial for achieving efficacy similar to that of commonly used sprayable insecticides.

Maintenance of low humidity is generally recommended to eliminate the risk of silverfish nuisance. However, this measure was designed for *L. saccharinum* and could not be effective enough for *C.* *longicaudatum* because of its low environmental requirements. Therefore, preventing the introduction of this species is difficult. The risk of contamination might be reduced by proper handling of incoming materials and rapid elimination of packages [18,54]. In the case of museums, libraries, and historic buildings, the IPM involves sealing the building against pest entry, adapting the micro-climate, maintaining high hygienic standards, quarantining all new and incoming objects, and regular monitoring of pest infestations with traps [21]. Nevertheless, a 100% reliable prevention will require costly and logistically challenging measures such as heat, cold, or freeze treatment of the entire moving load [18]. Therefore, further campaigns on this invasion, including the development of novel approaches for both prevention and control, are essential. In addition, pest controllers should be educated in order to correctly identify the species and establish proper management.

## 5. Conclusions

The study proved the monitoring of social platforms and citizen science might be useful tools to monitor the invasion of *C. longicaudatum*. On the other hand, these records have to be considered carefully due to probability of misidentification in certain cases. The collaboration between pest controllers and entomologists is thus highly needed. Based on the confirmed events throughout the case countries, the invasion of *C.* *longicaudatum* in Europe continues. Moreover, the data by citizen science study showed that the species distribution is most likely extremely under-recorded and *C. longicaudatum* is currently one of the common nuisance indoor pests. Moreover, the insulation and central heating providing a stable dry and warm environment, serving as a perfect environment for *C. longicaudatum* and is probably a reason of its shift northward. Similar conditions exist in museums and galleries; thus, an increasing number of new records are expected in regions where *C. longicaudatum* is or will be introduced. It follows from the above mentioned that the early identification and control of pests especially in museums and galleries, including *C. longicaudatum*, should be given high priority according to the importance of keeping safe art paintings and other valuable materials in collections for the future.

## Figures and Tables

**Table 1:** The public sources surveyed within monitoring the occurrence of *Ctenolepisma longicaudatum* in Europe.

Source	Abbreviations	Available from
European Network on Invasive Alien Species	NOBANIS	https://www.nobanis.org, 31 March 2021
Fauna Europea	-	https://fauna-eu.org/, 6 April 2021
Global Biodiversity Information Facility *	GBIF	https://www.gbif.org, 8 April 2021
iNaturalist *	-	https://www.inaturalist.org, 12 April 2021
Observation	-	https://observation.org, 12 April 2021
Pan-European Species directories Infrastructure	PESI	http://www.eu-nomen.eu, 18 March 2021
What’s Eating Your Collection	-	https://www.whatseatingyourcollection.com, 12 May 2021

* In the case of GBIF, the dataset “iNaturalist” was excluded in order to not double the number of observations gathered from this database.

**Table 2:** The reported occurrence of *Ctenolepisma longicaudatum* in Czechia in 2017–2021.

Locality	Municipality	Region	Date	Location	Source
1	Praha	Praha	10/2017	Warehouse *	NIPH ID
2	Praha	Praha	06/2018	Gallery *	NIPH ID
3	Kroměříž	Zlín	07/2018	flat	facebook.com
4	Plzeň	Plzeň	12/2018	warehouse	NIPH ID
5	Adamov	South Moravian	02/2019	flat	facebook.com
6	Praha	Praha	05/2019	flat	facebook.com
7	Orlová	Moravian-Silesian	09/2019	flat	facebook.com
8	Praha	Praha	10/2019	Warehouse *	NIPH ID
9	Klášterec nad Ohří	Ústí nad Labem	01/2020	flat	facebook.com
10	Dobrovíz	Central Bohemian	01/2020	warehouse	NIPH ID
11	Praha	Praha	02/2020	shopping mall	NIPH ID
12	Praha	Praha	04/2020	house	iNaturalist.org
13	Bohumín	Moravian-Silesian	07/2020	flat	facebook.com
14	Klecany	Central Bohemian	07/2020	pub. institute	iNaturalist.org
15	Brno	South Moravian	09/2020	flat	iNaturalist.org
16	Praha	Praha	11/2020	warehouse	NIPH ID
17	Tišnov	South Moravian	01/2021	Flat *	facebook.com
18	Ostrava	Moravian-Silesian	02/2021	flat	facebook.com
19	Jaroměř	Hradec Králové	02/2021	factory	facebook.com
20	Kopřivnice	Moravian-Silesian	03/2021	factory	NIPH ID

* Personal inspection of researchers from NIPH. NIPH: National Institute of Public Health Prague.

**Table 3:** The reported occurrence *Ctenolepisma longicaudatum* in the United Kingdom and Republic of Ireland in 2016–2021.

Locality	Town/City	Country	Date	Location	Source
1	London	England	06/2016	museum	whatseatingyourcollection.com
2	Antrim	N. Ireland	07/2016	unknown	NBN
3	London	England	09/2016	gallery	whatseatingyourcollection.com
4	London	England	09/2017	museum	whatseatingyourcollection.com
5	Quainton	England	09/2017	archive	whatseatingyourcollection.com
6	Southampton	England	10/2017	student acc.	Killgerm ID
7	Cork	Ireland	10/2017	domestic	Killgerm ID
8	Doncaster	England	10/2017	domestic	Killgerm ID
9	Leeds	England	11/2017	flats	Killgerm ID
10	Cork	Ireland	12/2017	apartments	Killgerm ID
11	London	England	01/2018	museum	whatseatingyourcollection.com
12	Birmingham	England	01/2018	museum	whatseatingyourcollection.com
13	London	England	01/2018	store	whatseatingyourcollection.com
14	London	England	04/2018	gallery	whatseatingyourcollection.com
15	Antrim	N. Ireland	05/2018	unknown	NBN
16	Gloucester	England	09/2018	archive	whatseatingyourcollection.com
17	Templepatrick	N. Ireland	09/2018	unknown	NBN
18–23	Birmingham	England	11/2018–10/2019	domestic	citizen science reports
24	London	England	01/2019	museum	whatseatingyourcollection.com
25	Leeds	England	01/2019	domestic	Killgerm ID
26	Aberdeen	Scotland *	02/2019	domestic	Killgerm ID
27	Portsmouth	England	05/2019	store	whatseatingyourcollection.com
28	Ashford	England	06/2019	office	Killgerm ID
29	Cheltenham	England	07/2019	domestic	Killgerm ID
30	Galway	Ireland	09/2019	medical center	iNaturalist.org
31	London	England	09/2019	shopping centre	iNaturalist.org
32	London	England	09/2019	archive	whatseatingyourcollection.com
33	London	England	09/2019	archive	whatseatingyourcollection.com
34	London	England	10/2019	archive	whatseatingyourcollection.com
35	Manchester	England	12/2019	student acc.	iNaturalist.org
36	Birmingham	England	01/2020	student acc.	iNaturalist.org
37	Chelmsford	England	02/2020	garden centre	iNaturalist.org
38	Gloucester	England	04/2020	domestic	iNaturalist.org
39	London	England	04/2020	archive	whatseatingyourcollection.com
40	Hartley	England	06/2020	domestic	Killgerm ID
41	Ballina	Ireland	07/2020	domestic	iNaturalist.org
42	Ipswich	England	07/2020	apartments	Killgerm ID
43	North London	England	07/2020	domestic	Killgerm ID
44	Royal Tunbridge Wells	England	07/2020	warehouse	iNaturalist.org
45	Taunton	England	08/2020	domestic	whatseatingyourcollection.com
46	Totnes	England	10/2020	domestic	iNaturalist.org
47	Brighton	England	11/2020	apartments for students	iNaturalist.org
48	Portsmouth	England	12/2020	unknown	iNaturalist.org
49	Brentford	England	12/2020	domestic	iNaturalist.org
50	South East London	England	12/2020	museum	Killgerm ID
51	Peterborough	England	12/2020	domestic	Killgerm ID
52	Manchester	England	01/2021	domestic	iNaturalist.org
53	Chiswick	England	01/2021	unknown	iNaturalist.org
54	Balbriggan	Ireland	01/2021	domestic	iNaturalist.org
55	North West London	England	01/2021	domestic	Killgerm ID
56	Southampton	England	02/2021	warehouse	iNaturalist.org
57	Milton Keynes	England	03/2021	warehouse	iNaturalist.org
58	South East London	England	03/2021	unknown	Killgerm ID
59	Canterbury	England	03/2021	domestic	Killgerm ID

NBN: National Biodiversity Network. * the first record of *Ctenolepisma longicaudata* in Scotland.

**Table 4:** The abundance of *Ctenolepisma. longicaudatum* in Europe based on online databases, namely, iNaturalist, GBIF, Observation.org, Fauna Europea, NOBANIS, and PESI and available literature sources.

	iNaturalist	GBIF	Observation	Fauna Europea	NOBANIS	PESI	Literature
Albania	3	0	0	present	0	0	0
Andorra	0	0	0	0	0	0	0
Austria	33	1	0	0	present	0	9 [16,31]
Azores	0	66 *	0	0	0	present	0
Belarus	0	0	0	0	0	0	0
Belgium	29	66 *	0	0	0	0	6 [10]
Bosnia and Herzegovina	0	0	0	0	0	0	0
Bulgaria	0	0	0	0	0	0	0
Canary Islands	4	0	0	present	0	present	0
Croatia	1	0	0	0	0	0	0
Cyprus	1	0	0	present	0	present	0
Czechia	3	0	0	0	0	0	1 [26]
Denmark mainland	80	0	0	0	0	0	2 [32]
Estonia	1	1 *	1		0	0	5 [28]
Faroe Islands	0	0	0	0	0	0	65 [27]
Finland	22	0	0	0	0	0	1 [33]
France	51	2	1	present	0	present	1 [34]
Germany	67	16 *	14	0	0	0	2 [35]
Greece	23	0	4	present	0	present	0
Greenland	0	0	0	0	0	0	0
Hungary	2	0	2	0	0	0	0
Iceland	1	0	0	0	0	0	0
Ireland	3	0	0	0	0	0	0
Italy	105	0	2	present	0	present	1 [36]
Kosovo	0	0	0	0	0	0	0
Latvia	0	0	0	0	0	0	0
Liechtenstein	0	0	0	0	0	0	0
Lithuania	2	0	0	0	0	0	0
Luxembourg	3	0	1	0	0	0	0
Madeira	4	2	0	present	0	present	0
Malta	0	0	0	present	0	0	0
Moldova	0	0	0	0	0	0	0
Monaco	0	0	0	0	0	0	0
Montenegro	0	0	0	0	0	0	0
Netherlands	68	134 *	0	0	present	0	34 [37]
North Macedonia	0	0	0	0	0	0	0
Norway	6	32 *	0	0	0	0	6788 [11,18]
Poland	4	0	0	0	0	0	1 [38]
Portugal mainland	62	8 *	1	present	0	present	0
Romania	0	0	0	0	0	0	0
Russian Federation	3	0	0	0	0	0	0
San Marino	0	0	0	0	0	0	0
Serbia	0	0	0	0	0	0	0
Slovakia	1	0	0	0	0	0	0
Slovenia	1	0	0	0	0	0	0
Spain mainland + Balears	95	2	7	present	0	present	12 [39,40,41]
Sweden	5	91 *	0	0	0	0	1 [42]
Switzerland	11	0	0	0	0	0	0
Turkey	6	0	0	0			
Ukraine	3	0	0	0	0	0	0
United Kingdom	16	0	0	0	0	0	2 [30,43] **
Vatican City	0	0	0	0	0	0	0

* Records with identical global positioning system coordinates were regarded as a single record.; ** the first record for Scotland was obtained within this study.

## Data Availability

Data are contained within the article.
